# The impacts of deacetylation prior to dilute acid pretreatment on the bioethanol process

**DOI:** 10.1186/1754-6834-5-8

**Published:** 2012-02-27

**Authors:** Xiaowen Chen, Joseph Shekiro, Mary Ann Franden, Wei Wang, Min Zhang, Erik Kuhn, David K Johnson, Melvin P Tucker

**Affiliations:** 1 National Bioenergy Center, National Renewable Energy Lab, 1617 Cole Blvd, Golden, CO 80127, USA; 2 Biosciences Center, National Renewable Energy Lab, 1617 Cole Blvd, Golden, CO 80127, USA

**Keywords:** Bioethanol, Pretreatment, Enzymatic Hydrolysis, Fermentation, Deacetylation

## Abstract

**Background:**

Dilute acid pretreatment is a promising pretreatment technology for the biochemical production of ethanol from lignocellulosic biomass. During dilute acid pretreatment, xylan depolymerizes to form soluble xylose monomers and oligomers. Because the xylan found in nature is highly acetylated, the formation of xylose monomers requires two steps: 1) cleavage of the xylosidic bonds, and 2) cleavage of covalently bonded acetyl ester groups.

**Results:**

In this study, we show that the latter may be the rate limiting step for xylose monomer formation. Furthermore, acetyl groups are also found to be a cause of biomass recalcitrance and hydrolyzate toxicity. While the removal of acetyl groups from native corn stover by alkaline de-esterification prior to pretreatment improves overall process yields, the exact impact is highly dependent on the corn stover variety in use. Xylose monomer yields in pretreatment generally increases by greater than 10%. Compared to pretreated corn stover controls, the deacetylated corn stover feedstock is approximately 20% more digestible after pretreatment. Finally, by lowering hydrolyzate toxicity, xylose utilization and ethanol yields are further improved during fermentation by roughly 10% and 7%, respectively. In this study, several varieties of corn stover lots were investigated to test the robustness of the deacetylation-pretreatment-saccharification-fermentation process.

**Conclusions:**

Deacetylation shows significant improvement on glucose and xylose yields during pretreatment and enzymatic hydrolysis, but it also reduces hydrolyzate toxicity during fermentation, thereby improving ethanol yields and titer. The magnitude of effect is dependent on the selected corn stover variety, with several varieties achieving improvements of greater than 10% xylose yield in pretreatment, 20% glucose yield in low solids enzymatic hydrolysis and 7% overall ethanol yield.

## Background

The development of cost-competitive cellulosic biomass conversion processes is highly dependent on the realization of high unit operation yields and high overall process yields. To achieve such a goal, changes within each unit operation must be evaluated carefully within the context of the entire process. While high xylan-to-xylose yields are possible by many pretreatment technologies, fermentation inhibitors generated within pretreatment processes may lower fermentation yields. One known inhibitor is acetic acid that is formed by cleavage of covalently bonded acetyl groups from the xylan backbone during dilute acid pretreatment. Corn stover has an ultrastructure similar to other varieties of lignocellulosic biomass wherein hemicellulose surrounds and interconnects the cellulosic fibers, helping to provide rigidity. Typically corn stover is composed of approximately 37% cellulose, 21% xylan, 18% lignin and 3% acetyl groups on a dry weight basis [1]. Acetyl groups are present in an acetate to xylose ratio of approximately 2:5, and they are covalently bonded to xylan by ester bonds at the 2 and 3 carbon positions in the pyranose ring [2].

Acetyl groups can be liberated during hydrolysis of the xylan backbone and the removal of side chains during pretreatment and enzymatic hydrolysis [3]. However, after dilute acid pretreatment, a significant portion of the original xylan remains as oligomers, with many of the oligomers containing acetyl side chains, thus lowering potential yields and also inhibiting enzyme activity in the subsequent enzymatic saccharification step [4].

While some reports in the literature have addressed the issue of recalcitrant xylooligomers with the inclusion of accessory enzymes such as acetyl xylan esterases (AXE) [5], accessory enzyme activity was found to be sensitive to the background concentration of sugars present, and the slurries required dilution in order for the enzymes to be effective. Other research groups have found that acetate in solution depresses enzymatic hydrolysis of oligomers in hydrolyzate [2,5-11]. Alternatively, Mitchell *et al*. found that removing acetyl groups prior to pretreatment dramatically improved the cellulose and xylan digestibilities by two to three and five to seven times, respectively, compared to results from untreated corn stover [12].

After enzymatic saccharification, liberated acetate continues to act as a fermentation inhibitor for many microorganisms, including *Zymomonas mobilis *CP4 (pZB5) and *E. coli *KO11 [3,8,13,14]. Maiorella *et al*. postulated that soluble acetate disrupts the transportation of nutrients, such as phosphate, across the cell membrane [15].

Deacetylation and removal of acetic acid prior to pretreatment have the potential to decrease otherwise high feedstock variability by reducing the neutralization capacity of the feedstock through the removal of water soluble extractives and ash, and it also minimizes the buffering capacity of the residual acetic acid in the feedstock [16,17]. There is also evidence that removing acetate can improve xylose and glucose utilization in fermentation [18].

Acetyl groups esterified to the hemicellulosic structure of corn stover biomass have been shown to increase biomass recalcitrance and, when solubilized during pretreatment and enzymatic hydrolysis, to inhibit fermentation. Removal of acetic acid solubilized during pretreatment and enzymatic hydrolysis is problematic due to the difficulty of introducing sophisticated separations processes at this step [19]. Alternatively, the application of a dilute alkaline extraction step prior to pretreatment has been shown to remove up to 75% of acetyl groups from raw corn stover [20]. Removal of potential inhibitors from biomass before further processing has been shown to improve pretreatment and enzymatic hydrolysis yields as well as to reduce hydrolyzate toxicity in fermentation [18,20].

The current work demonstrates the impact of removing acetate (deacetylation) by dilute alkaline extraction prior to pretreatment on the entire corn stover-to-ethanol process, which results in improved monomeric sugar yields in low acid, low-severity dilute acid pretreatment, increased cellulose and xylan digestibility in high solids (> 20 wt % slurries) enzymatic saccharification, and increased hydrolyzate fermentability during fermentation.

## Results and discussion

### The effect of deacetylation on corn stover composition

During the alkali extraction, approximately 20% of the of the initial corn stover mass was extracted through the removal of sucrose, acetate and other miscellaneous extractives. Following deacetylation, the weight of all of the corn stover samples decreased by roughly 20%, from 9.3 kg of oven dry (O.D.) weight to 7.2 kg for Kramer 34M95, 7.3 kg for the INL corn stover and Kramer 33A14, and 7.5 kg for Kramer 33B51. The weight loss is due to the extraction of most of the sucrose (3 to approximately 5 wt % based on initial weight), acetate (2 to approximately 3 wt %), and other extractives (6 to approximately 10 wt %), plus a smaller amount of xylan (< 1 wt %), glucan (< 0.5 wt %), and lignin (2 to approximately 4 wt %).

Table 1 shows that the acetate content of the deacetylated corn stover was about one-third that of the control (acid-impregnated only) corn stover. The deacetylated corn stover lignin content also was about 1% to approximately 3% lower than that of the control. Thus, the glucan and xylan content increased due to the multi-component extraction.

**Table 1:** Compositional analysis of acid-impregnated corn stover with and without deacetylation

Sample ID		% Ash	% Lignin	% Glucan	% Xylan	% Galactan	% Arabinan	% Acetate	Total %
33A14 (Kramer)	Without Deacetylation	3.5	18.1	42.3	24.8	1.4	3.7	2.9	96.7
34M95 (Kramer)		1.7	17.8	40.8	25.4	1.3	3.3	3.8	94.2
33B51 (Kramer)		2.1	18.9	41.1	23.5	1.3	3.1	4.0	94.0
INL		5.1	20.2	37.7	24.0	1.4	3.2	3.1	94.7

33A14 (Kramer)	With Deacetylation	3.5	15.6	42.8	25.4	1.6	3.8	1.3	94.1
34M95 (Kramer)		1.0	17.4	43.1	28.3	1.7	3.0	1.2	95.7
33B51 (Kramer)		1.4	17.3	45.5	24.6	1.3	3.4	1.3	95.1
INL		4.5	17.4	44.0	26.0	1.4	2.9	1.0	97.2

### Effects of deacetylation on xylose conversion during pretreatment

**Figure 1:**
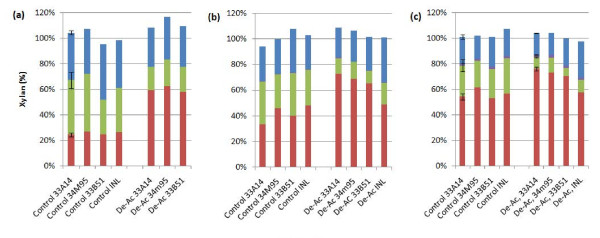
**Xylose yield during pretreatment at residences times of (a) 5 minutes, (b) 10 minutes, and (c) 20 minutes; **%solubilized xylose monomers (red); % solubilized xylose oligomers (green); % xylose converted into furfural (purple); % xylan remaining in pretreated solids (blue).

Pretreatment experiments were carried out at 150°C, at approximately 45% initial solids loading, for 5, 10, and 20 minutes. The sulfuric acid loading was approximately 8 mg/g of biomass, as measured by titration, for both control and deacetylated acid impregnated corn stover feedstocks. The initial pH was approximately 1.3, and the final pH ranged from 1.7 to approximately 2.0 depending on residence time in the reactor. The higher pH values measured at the longer residence times were the result of increased steam condensate accumulation. Figure 1 shows the xylan mass distribution in the post-pretreatment solids and liquid. Different corn stover varieties were found to have different degrees of xylan solubilization and depolymerization, and conversion yields responded differently to deacetylation. As shown in Figure 1, corn stover varieties from the Kramer farm were more sensitive to deacetylation. Xylose monomer (XM) yields are statistically greater for deacetylated corn stover feedstocks when compared to non-deacetylated feedstocks. For example, XM yields for non-deacetylated 34M95 are 27%, 46% and 62% for 5, 10, and 20 minutes, respectively, while for deacetylated 34M95 the corresponding XM yields are 63%, 69% and 73%. Depolymerization of xylan to xylose in acid pretreatment involves two reactions: A) hydrolysis of xylosidic bonds, and B) hydrolysis of ester bonds between xylose subunits and acetyl groups. Approximately 40% of the xylan in corn stover is acetylated, suggesting that 40% of xylose monomer formed in pretreatment requires both reactions to take place. Following dilute alkali deacetylation, approximately two-thirds of the acetyl groups have been removed before pretreatment, resulting in approximately 87% of the xylan-to-xylose monomer formation requiring reaction A, while the remaining 13% of XM formation requires both reactions A and B. Lower xylose monomer yields from pretreatment of non-deacetylated corn stover feedstocks suggest that reaction B is the rate limiting step for xylose monomer formation at the pretreatment conditions experimented [20].

Increasing the pretreatment residence time reduces the difference in XM yield between control and deacetylated corn stover. For example, the differences in xylose monomer yields between control and deacetylated 34M95 feedstock are 36% at 5 minutes (27% for control vs. 63% for deacetylated), 23% at 10 minutes (46% for control vs. 69% for deacetylated), and 11% at 20 minutes (62% for control vs. 73% for deacetylated), showing a decreasing trend of deacetylation effect with increasing pretreatment severity. This result may be explained by a higher fraction of solubilized xylan being deacetylated by acid de-esterification during higher severity pretreatments. Table 2 shows that solubilized acetylated xylan oligomers are increasingly deacetylated during dilute acid pretreatment at the conditions tested.

**Table 2:** Effects of deacetylation and pretreatment residence times on acetate release from solubilized xylooligomers

a	Kramer 34M95	Pretreatment residence times (min)	Free acetate/xylose monomer (mol/mol)	Free acetate/total solubilized xylose monomer (mol/mol)*	Bound acetate/xylose oligomer	Total acetate/total solubilized xylan** (mol/mol)
	Control	5	0.20	0.20	0.46	0.36
		10	0.21	0.21	0.57	0.36
		20	0.27	0.26	0.72	0.38
	Deacetylated	5	0.04	0.04	0.27	0.10
		10	0.07	0.07	0.33	0.11
		20	0.10	0.09	0.34	0.12

**b**	**Kramer 33B51**	**Pretreatment residence times (min)**	**Free acetate/xylose monomer (mol/mol)**	Free acetate/total solubilized xylose monomer (mol/mol)*	**Bound acetate/xylose oligomer**	Total acetate/total solubilized xylan** (mol/mol)

	Control	5	0.17	0.17	0.45	0.34
		10	0.19	0.19	0.55	0.35
		20	0.25	0.25	0.69	0.39
	Deacetylated	5	0.05	0.04	0.30	0.11
		10	0.08	0.07	0.34	0.11
		20	0.11	0.10	0.40	0.14

*total solubilized xylose monomer consists of solubilized xylose in monomer form and in degraded form (furfural)

**total solubilized xylan consists of xylose monomer, xylose oligomer and furfural

In the liquor phase of pretreated slurry, acetate is present in two forms: 1) free acetate/acetic acid, released from the xylan; and 2) acetyl groups covalently bound to the dissolved xylan oligomers. Table 2a shows that the molar ratio of total acetate over total solubilized xylan for control Kramer 34M95 increased from 0.37 to 0.39, showing that the solubilization of xylan results from a random cleavage of the xylan chain because native xylan structure in variety 34M95 has an acetyl-to-xylose ratio of 0.40. This result is similar to that found using the deacetylated corn stover feedstock. The deacetylated Kramer 34M95 prior to pretreatment was found to have an acetyl-to-xylose ratio of 0.13, which is very close to the acetyl-to-xylose ratio found for the solubilized xylan in the liquor phase, as shown in Table 2a. However, much lower free acetate-to-xylose monomer ratios in both the control and deacetylated pretreated Kramer 34M95 liquors show that acetate removal during dilute-acid pretreatment under acidic conditions lags behind xylan hydrolysis. Longer pretreatment residence times tend to cleave additional acetyl groups from dissolved xylooligomers, while the xylooligomer-to-xylose reaction slows down and eventually reaches equilibrium, resulting in a higher free acetate-to-xylose monomer ratio. It is noticeable that the increasing acetate to xylose monomer ratio is not caused by the degradation of xylose to furfural as residence time increased. As shown in Table 2a, degradation of xylose monomers are taken into account for the total solubilized xylose monomer, which does not significantly change the free acetate to xylose monomer ratio. The similar increasing trends of free acetate over xylose monomer ratio were found for all the other corn stover lot. Table 2b shows similar observations for Kramer 33B51 lot. Among the three Pioneer varieties of corn stover harvested from the Kramer farm, 33B51 was found to have lower xylose monomer yields and total solubilized xylan yields at the same pretreatment conditions. Pioneer variety 33A14 was found to have the highest monomeric and total solubilized xylan yields. Common among all the Pioneer varieties of corn stover from the Kramer farm is that deacetylation led to higher xylose monomer yields, higher overall solubilized xylan yields and lower xylooligomer yields. However, the INL corn stover feedstock showed only a modest increase in xylose monomer yields (1% to approximately 2%), although deacetylation resulted in 10% to approximately 15% lower xylooligomer yields and 7% to approximately 10% lower overall solubilized xylan yields. The reason for the decreased xylan-to-soluble xylose yields is not clear. Because the INL corn stover, prior to any processing, has a lower acetate content but a much higher ash and lignin content compared to Pioneer varieties of corn stover from the Kramer farm, adjustment in deacetylation conditions may be needed in order to optimize xylan-to-soluble xylose yields in pretreatment. The difference in xylose yield of the INL corn stover compared to that of the Kramer lots highlights the effect that feedstock variability can have on process yields. The INL corn stover remained on the field for a substantial period of time before being moved to dry storage; this likely contributes to the greater recalcitrance of the INL stover compared to the Kramer lots, which were harvested then immediately dry-stored [21,22]. Further research should be conducted to provide insight into the effects of feedstock variability within a given plant species on pretreatment component yields.

### The effects of deacetylation and pretreatment on low solids enzymatic hydrolysis

**Figure 2:**
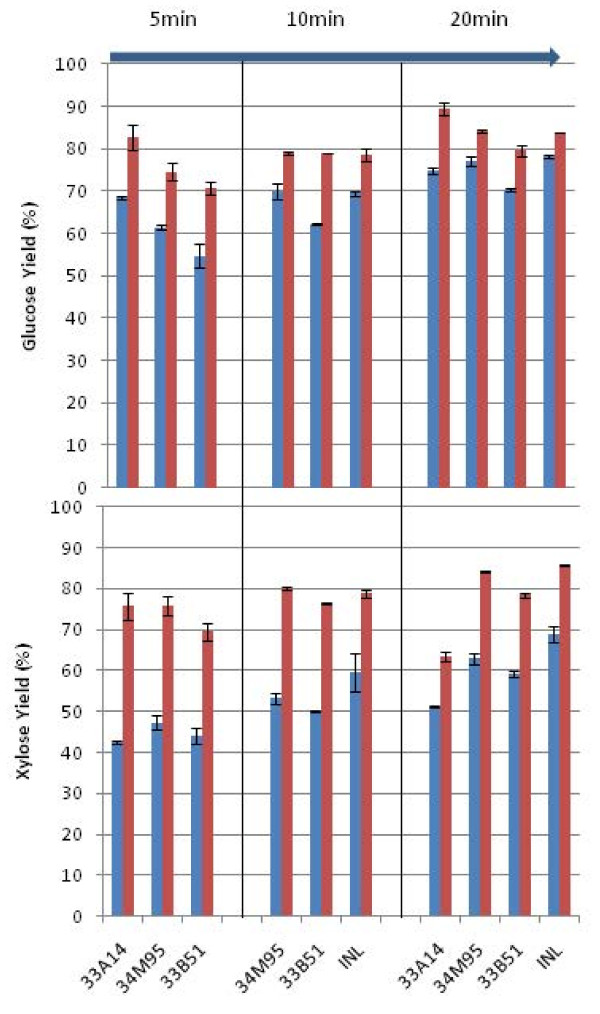
**Glucose and xylose yields during low solids enzymatic hydrolysis (1% glucan loading); **Control PCS (blue) Deacetylated PCS (red).

Enzymatic hydrolysis under low solids conditions (1 wt % cellulose loadings) was performed on the washed pretreated corn stover substrates as described previously. Figure 2 shows glucose and xylose yields after 168-hour digestions. The sugar yields were calculated based on the compositional analysis of pretreated solids. Enzymatic digestion yields of both control and deacetylated corn stover pretreated at 150°C, 0.5 wt % H_2_SO_4_, and from 5 minutes to 20 minutes residence times are shown.

For a five-minute pretreatment, the glucose yields for control samples range from 57% to 71% for Kramer corn stover. Pioneer variety 33B51 was found to be the most recalcitrant with respect to enzymatic digestion, probably because of the decreased amount of xylan solubilized in pretreatment. The glucose yields were all improved by an average of 15% using deacetylated substrates. Deacetylation also significantly improved xylose yields in enzymatic digestions, from an average of 43% for control substrates to 74% for deacetylated substrates.

Higher glucose and xylose yields were found with increasing pretreatment residence times, especially for the control substrates. Glucose yield for the Pioneer variety 34M95 control was found to increase from approximately 64% for 5 minutes to approximately 72% for 10 minutes and to approximately 79% for 20 minutes. Similar glucose yield improvements were found with the control sample of 33B51 by increasing pretreatment residence times; glucose yield increased from approximately 57% at 5 minutes to approximately 64% at 10 minutes and to approximately 72% at 20 minutes. The same gradual improvement was also observed in xylose yields. For 34M95, xylose yields improved from approximately 47% to approximately 63% from pretreatment at 5 minutes to 20 minutes, respectively. Deacetylation significantly improves the digestibility of pretreated corn stover (PCS). Glucose yields improved by an average of approximately 12%, while xylose yields improved by an average of approximately 23%. The significant yield improvement demonstrates the importance of removing acetyl groups from xylan prior to enzymatic hydrolysis.

Table 3 shows the acetyl-to-xylose molar ratio in the solids before pretreatment, in the residual solids after pretreatment, and in the residual solids after enzymatic hydrolysis for native and deacetylated corn stover feedstocks. The acetyl-to-xylose molar ratio represents the degree to which xylose units in xylan are esterified with acetyl groups. Native corn stover after acid impregnation was found to have an acetyl-to-xylose molar ratio of approximately 0.45, which corresponds to approximately two out of five xylose units on the xylan backbone being covalently bonded with acetyl groups. For deacetylated corn stover, this ratio drops to approximately 0.14, which is equivalent to one acetyl group per seven xylose units. After pretreatment, the ratios for both the control and deacetylated corn stover are not significantly increased, which is in agreement with the liquid analysis shown in Table 3, indicating that the acetyl groups are equally distributed on the xylan backbone and that acid hydrolysis of xylan is a random chain scission. However, after enzymatic hydrolysis, the acetyl-to-xylose ratio is significantly higher: 0.8 to approximately 0.9 (approximately 4:5) for native corn stover substrates and 0.6 to approximately 0.7 (approximately 3:5) for deacetylated corn stover. The higher acetyl-to-xylose ratio indicates that the enzymatic hydrolysis is highly selective, leaving the highly acetylated xylan in the residual solids. Apparently, enzymes in the commercial cocktails prefer to hydrolyze xylan that is sparsely populated with acetyl groups, leaving the acetylated xylan in the solids. The result is a highly acetylated recalcitrant xylan in the residual solids. These results suggest that the current enzyme mixtures are not able to hydrolyze the highly acetylated xylan effectively. Additional accessory enzymes, such as AXE (acetyl xylan esterase), may be needed to further improve xylose yields in enzymatic saccharification. The higher xylose yields observed for deacetylated corn stover suggest that removing residual xylan in solids could possibly improve the accessibility of cellulose to enzyme, which would lead to higher glucose yields.

**Table 3:** Acetyl-to-xylose molar ratio

	Before Pretreatment			After Pretreatment (20 min residence time)			After Enzymatic Hydrolysis (168 h residence time)		
	
	34M95	33B51	INL	34M95	33B51	INL	34M95	33B51	INL
Native	0.46	0.52	0.40	0.50	0.50	0.51	0.81	0.83	0.88
Deacetylated	0.13	0.16	0.12	0.19	0.25	0.12	0.56	0.59	0.66

### Hydrolyzate fermentability analysis

To test the effect of acetate removal prior to pretreatment on fermentation yields, we performed higher solids (25% solids) enzymatic saccharification experiments using unwashed solids. There are several reasons for selecting high solids, whole slurry enzymatic hydrolysis. First, higher solids enzymatic hydrolysis results in higher sugar concentrations in fermentation, requiring less energy demand for the distillation process for ethanol purification. Humbird *et al*. reported that the minimum solids loading occurred between 15% and 20% total solids in saccharification and fermentation based on their techno-economical analysis [23]. Also, washing pretreated solids is difficult because the fines produced require a solid/liquid separation step, which is expensive when implemented at the industrial scale. Lastly, using a higher solids loading leads to higher acetate concentrations in hydrolyzate, which may lead to greater inhibition of fermentation by *Z. mobilis*. Therefore, enzymatic hydrolysis on whole slurry was carried out at a total solids content of 25%, which includes about 18% insoluble solids.

**Figure 3:**
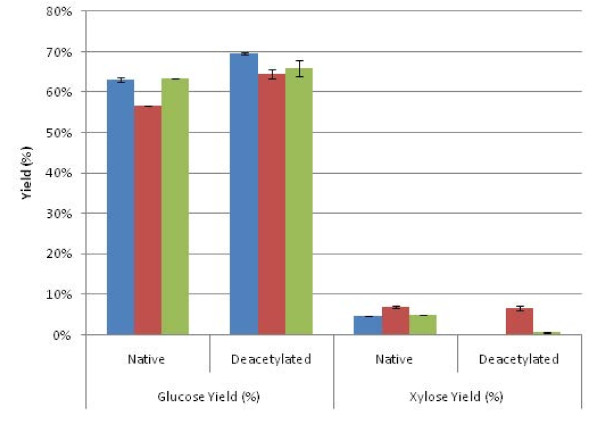
**Whole slurry enzymatic hydrolysis yields (25 wt % solids)**. Kramer 34M95 (blue); Kramer 33B51 (red); INL (green).

Figure 3 shows that glucose and xylose yields in high solids enzymatic saccharification were significantly lower compared to enzymatic hydrolysis yields using washed low solids concentrations as shown in Figure 2. This is due to the following: 1) enzyme mixing limitations of high solids slurries; and 2) inhibition of the enzymes due to high xylose/xylooligomer concentrations in the background sugars from pretreatment [4,11]. The liquor phase in the whole slurry contains 70 to approximately 80 g/L of xylose, which apparently strongly inhibits the xylanase during the digestion of the residual xylan in the solids. Hence, the xylose yields during high solids enzymatic hydrolysis for both control and deacetylated corn stover are under 10%, leading to a much lower glucose yield. The lower xylan removal also decreases the effect of deacetylation because the improved enzyme accessibility gained by removing xylan is significantly reduced. As shown in Figure 3, the glucose yield for deacetylated corn stover is only 2% to approximately 9% higher than that of control corn stover.

After saccharification, a toxicity analysis was performed on the hydrolyzates. Whole slurries were analyzed using two assays developed for toxicity analysis: growth assays using the Bioscreen C [24] and mini-fermentations assays, both using *Z. mobilis *8b as the biocatalyst. Because growth assays are performed using spectrophotometric analysis, solids are removed by centrifugation and the supernatant liquor is filtered. Due to the opaqueness of the supernatant liquor from high solids slurries, samples were diluted to 10 wt % of the pretreatment slurry with the addition of 10 g/L yeast extract, 2 g/L K_2_HPO_4_, water, and sugars, while normalizing the concentrations for both glucose and xylose.

**Figure 4:**
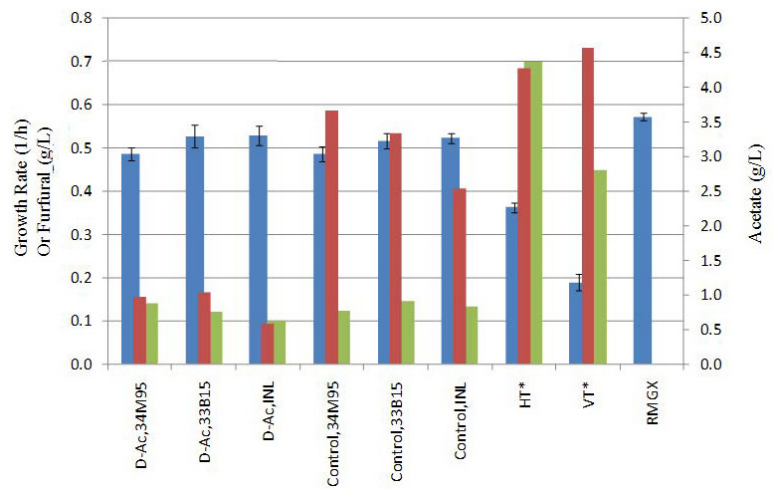
**Growth rates of *Z. mobilis *8b in filtered whole slurries at 10% total solids equivalent plotted with inhibitor concentrations**. Growth rate (blue); Acetic acid (red); Furfural (green).

Growth rates of *Z. mobilis *8b in whole slurries from deacetylated 34M95, 33B15, and INL feedstocks were compared with their respective non-deacetylated controls, as shown in Figure 4. In addition, two hydrolyzate liquor samples obtained from higher severity pretreated corn stover were also tested for toxicity in this study for comparison purposes. VT refers to corn stover pretreated in a pilot scale vertical reactor at 190°C for one minute in 3% sulfuric acid. The details of the reactor and its relevant acid pretreatment process have been reported by Shell *et al. *[25]. HT refers to corn stover pretreated in the pilot scale horizontal reactor for 158°C for five minutes using 2% acid (Nagle, Kuhn, Shekiro, unpublished data). Pure sugar fermentations were conducted as positive controls for both growth and mini-fermentation assays. We have noted slightly different growth rates for *Z. mobilis *8b when grown in different sugar concentrations. Other laboratories have also noted specific growth rates to change at high glucose concentrations [26]. For this reason, glucose and xylose levels were normalized based on the concentrations of sugars in samples containing the maximum levels.

Growth rates for deacetylated and control slurry samples are not significantly different at the 10% total solids equivalent level; both are approximately 15% to 20% lower than growth rates observed with the pure sugar controls. Furfural concentrations in both control and deacetylated 10% total solid slurries are very similar (approximately 0.1 g/L) and are 78% to 86% lower than concentrations observed in the higher severity pretreated hydrolyzates experimented (0.45 to 0.7 g/L), which causes significantly more growth inhibition. Growth rate inhibitions at 0.1 g/L furfural are predicted to be minimal (< 5%) for *Z. mobilis *8b when no other inhibitors are present [24]. Likewise, the acetate levels of < 5 g/L are predicted to have minimal impact on growth in the absence of other inhibitors [24]. Therefore, if the toxicity contribution is only from acetate or furfural, one would not expect to see much difference in fermentation yields between deacetylated and control samples at this solids loading, which was observed.

**Figure 5:**
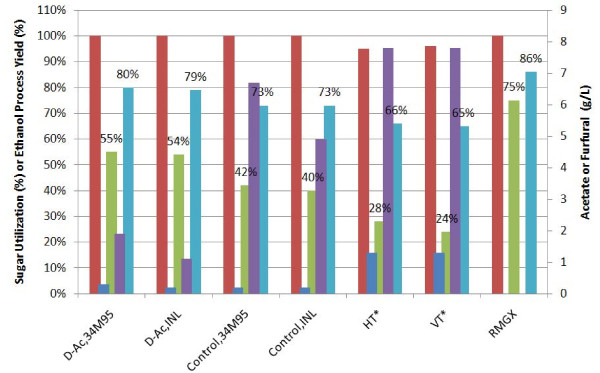
**Whole slurry xylose conversion yields from *Z. mobilis *8b at 18.5% total solids equivalent plotted with inhibitor concentrations**. %glucose utilized (red); %xylose utilized (green); EtOH yield (cyan); Acetate (purple); Furfural (blue).

Mini-fermentations were conducted at the process relevant condition of 18.5% total equivalent solids after normalizing for glucose (83 g/L) and xylose (56 g/L), medium, and inoculum. Fermentations were carried out with *Z. mobilis *8b at an inoculum loading of 1.0 (OD_600_) at 33°C. In Figure 5, process ethanol yields and sugar utilization consumed were plotted along with the inhibitor concentrations of acetate and furfural. Glucose utilization was complete for all samples except for the higher severity samples, HT and VT, where approximately 5% of the initial glucose remained post fermentation. In pure sugars, *Z. mobilis *was able to ferment 75% of the initial xylose (without pH control). Xylose conversion yields in deacetylated samples were approximately 10% higher when compared to control samples and resulted in approximately 7% higher ethanol yields. When compared to hydrolyzates generated from higher severity pretreatment conditions, VT and HT, xylose utilization was approximately 25% greater, resulting in approximately 15% higher ethanol process yields.

## Conclusions

**Figure 6:**
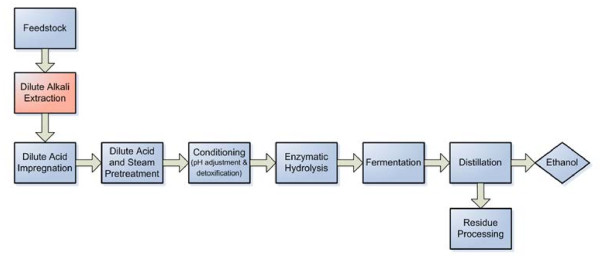
**Modified bio-ethanol platform by adding a dilute alkali extraction (deacetylation) prior to acid pretreatment**.

The deacetylation step is a proposed modification of a dilute acid pretreatment/enzymatic saccharification/fermentation bioethanol process, as shown in Figure 6. The dilute alkali extraction step before acid pretreatment removes approximately two-thirds of the acetate in corn stover. When the yields are compared to yields for control corn stover feedstock, the deacetylated corn stover has higher monomeric xylose yields and lower oligomeric xylose yields after dilute-acid steam pretreatment, while the total amount of xylan solubilized is similar between control and deacetylated corn stover. The acetyl-to-xylose ratio in the liquid phase of dilute-acid pretreatment hydrolyzates indicates that the deacetylation reaction is the rate-limiting step for xylose monomer formation during dilute-acid pretreatment. The effect of deacetylation also differs for different corn stover varieties. The three Kramer corn stover varieties responded similarly to deacetylation, while deacetylated INL corn stover showed a reduction in xylose oligomer yield and total solubilized xylan after pretreatment. Hence, different reaction mechanisms may exist for the INL corn stover during dilute alkali extraction.

Deacetylation also significantly improved corn stover digestibility during low solids enzymatic hydrolysis using washed, pretreated solids. The glucose yields were improved by an average of 12%, while xylose yields were improved by an average of 23%. The significant improvement shows the importance of removing acetyl groups from xylan prior to enzymatic hydrolysis. The elevated acetyl-to-xylose ratio in enzymatic hydrolyzed solid residue indicates that enzymes preferentially hydrolyze xylose that lacks acetyl side-chains, leaving much of the acetylated xylose in the solids. Although the addition of AXE (Acetyl Xylan Esterase) will also improve xylose yield by cleaving acetyl groups from xylan backbone enzymatically, the cost of AXE would significantly increase the operational costs. In addition, the released acetate groups by AXE will end up in fermentation broth and thus decrease the ethanol yields.

High solids enzymatic hydrolysis using unwashed, pretreated solids was performed in order to determine 1) the deacetylation effect of high solids, whole slurry enzymatic hydrolysis; and 2) the toxicity of acetate in enzymatic hydrolyzates. The deacetylation effect is reduced due to xylanase inhibition at high xylose concentrations. However, the removal of acetyl groups improved xylose utilization by 10% in fermentation, leading to a 7% higher ethanol yield.

In summary, not only does deacetylation show significant improvement on glucose and xylose yields during pretreatment and enzymatic hydrolysis, but it also significantly reduces hydrolyzate toxicity during fermentation, thereby improving ethanol yields and titer.

## Methods

### Materials

Four hybrid varieties of corn stover were investigated. The Pioneer varieties 33A14, 34M95 and 33B51 were harvested from the Kramer farm in Wray, Colorado. The fourth type of corn stover, INL, was harvested in 2008 by Dr. Neal Yancey at Idaho National Laboratory (INL) from a farm near Hugoton, Kansas, following a test of harvesting methods. The compositional analysis results for the four corn stover varieties are shown in Table 4. Each corn stover lot was tub ground at the farm and then further milled at NREL through a Mitts & Merrill rotary knife mill (model 10 × 12, Saginaw, MI, USA) to pass a 1/2-inch (12.7 mm) rejection screen. All Kramer corn stover lots were stored in a dry place immediately after harvest. INL corn stover was left on the field for six months post harvest then stored in a dry place. Therefore, the INL corn stover lot had a lower sucrose content than the Kramer corn stover lots. The INL corn stover also had the lowest acetyl content and the highest lignin content of the group, as shown in Table 4.

**Table 4:** Compositional analysis of corn stover feedstocks

Sample Description	%Total Ash	%Structural Inorganics	%Non-structural inorganics	% Sucrose	%Water Extractable Others	%Ethanol Extractives	% Lignin	% Glucan	% Xylan	% Galactan	% Arabinan	% Fructan	% Acetyl	Total %
34M95 (Kramer)	2.8	1.4	1.4	5.6	6.5	2.3	13.2	34.2	22.7	1.3	3.1	0.5	3.4	95.5
33B51 (Kramer)	4.6	1.8	2.8	5.6	10.2	2.5	12.4	34.4	18.7	1.1	2.7	0.4	3.0	95.6
33A14 (Kramer)	6.1	4.8	1.3	3.5	10.8	2.2	11.6	33.1	20.6	1.5	3.9	0.0	2.8	97.7
INL (Hugoton)	5.7	3.9	1.9	0.4	5.0	2.6	16.0	35.7	21.3	1.6	3.4	0.5	2.6	94.8

### Deacetylation

Deacetylation of each corn stover lot was performed by dilute alkali extraction in a Recirculating Atmospheric Pressure Impregnation (RAPI) system as described by Tucker [27]. Approximately 10 kg of dry (approximately 93 wt %) 1/2-inch milled corn stover was loaded into a Hastelloy C-276 wire mesh (40 mesh screen) basket and immersed in the recirculating bath containing 120 L of 0.4 wt % NaOH (0.1 M) solution at 70°C for three hours. The initial pH was approximately 12. After extraction, the excess alkali solution was drained and a liquid sample was retained for pH, acetate and sugar concentration analysis. The solid sample was weighed and measured for percent total solids.

### Acid impregnation

Acid impregnation was performed using the same system described [27]. Approximately 120 L of warm (48° to 50°C), 0.5 wt % H_2_SO_4 _solution was prepared in a recirculation tank. A Hastelloy C-276 wire mesh (40 mesh screen) basket was loaded with 10 kg of 1/2-inch milled corn stover (approximately 93% solids) and immersed into the warm (48° to 50°C) recirculating dilute sulfuric acid bath for two hours. After acid impregnation, the feedstock in the basket was drained of excess acid solution to approximately 16% to approximately 18% solids and loaded into the mold of a hydraulic dewatering press, where the acid impregnated feedstock was pressed to approximately 45% solids.

### Steam explosion

The NREL 4-L steam gun reactor was used for all pretreatment experiments. The NREL steam gun is a 4-L steam-explosion reactor equipped with a jacket, a 4-in (10-cm) ball valve at the top for loading biomass, a 2-in (5-cm) ball valve at the bottom for discharging the contents of the reactor, two steam-injection ports near the top and bottom, and K-type thermocouples inserted near the top and bottom for reactor-temperature measurements. The reactor is made of Hastelloy C-22 TM to resist corrosion. The reactor temperature is controlled at the desired value using a pressure-control valve to regulate the steam-supply pressure. At the beginning of each experiment the reactor is preheated to the desired operating temperature by injecting steam into the jacket and cycling steam repeatedly through the reactor. The reactor is then loaded with the desired amount of pre-processed feedstock and quickly heated (approximately 5 to 10 sec) via direct steam injection to the reaction temperature. After the desired pretreatment time the steam is shut off and the bottom ball valve is quickly opened (< 1 second), explosively discharging the pretreated solids into a 55 gallon nylon HotFill (CDF Corporation, Plymouth, MA, USA) bag inside a 200-L flash tank. The bag is removed from the flash tank, labeled, sealed and stored at 4°C until ready for analysis.

The steam explosion reactor was pre-warmed to pretreatment temperature (150°C) and then loaded with 750 g of acid impregnated corn stover (approximately 45% solids). Direct steam injection brings the feedstock quickly to the reaction temperature (150°C) in less than 15 seconds. Pretreatments were carried out at the conditions of 150°C, 0.5 wt % H_2_SO_4_, and at 5, 10, and 20 minutes residence times.

### Enzymatic hydrolysis

Novozymes (Raleigh, NC, USA) cellulase, Cellic CTec2, and hemicellulase, Cellic HTec2, were used in the current study. The Novozymes enzyme preparations are a proprietary mixture of various enzyme activities. The enzyme loadings were calculated on a protein basis for 20 mg CTec2 (16FPU) and 2 mg HTec2 per gram of cellulose. The protein concentrations of the two commercial enzyme preparations were measured by the BCA assay (Thermo Scientific Pierce Biotechnology, Rockford, IL, USA) calibrated versus standard curves derived from bovine serum albumin standards supplied with the kits.

For washed solids enzymatic hydrolysis, the pretreated slurries were repeatedly washed with DI water in centrifuge bottles at 10,000 rpm by centrifugation and decanting until the xylose concentration was measured below < 0.01 g/L by a Yellow Springs Instrument 7100 MBS (Yellow Springs, OH, USA) calibrated versus supplied standards; usually six cycles were required. Enzymatic hydrolysis under dilute conditions (approximately 2% solids) was performed in 125 mL Erlenmeyer flasks containing 2.5 mL 50 mM citrate buffer, pH 4.8, 2.5 mg tetracycline, and enzyme, at a glucan loading of 1.0% (w/v). Enzymatic hydrolysis conditions were 150 rpm at 50°C.

### Hydrolyzate preparation for fermentability test

The pretreated corn stover samples were subjected to whole slurry enzymatic hydrolysis to prepare the hydrolyzate for the fermentability test. The unwashed slurry was adjusted to a pH of 5.0 by successive additions of small amounts of concentrated ammonium hydroxide (29 wt %) and mixing. No citrate buffer was added because of the inhibition of citrate ions to growth and fermentation. Saccharifications were carried out in 125 mL wide-mouth polypropylene roller bottles (Thermo Fisher Scientific, Inc., Waltham, MA, USA) with 60 g of pH-adjusted slurries at 25% total solids. The roller bottle saccharification reactors employ gravitational tumbling as the mixing mechanism to homogenize solids by horizontally rotating the reaction vessels at 4 rpm on a three-deck roller apparatus for mini bottles (Wheaton Industries Inc., Millville, NJ, USA) [28]. The roller apparatus was placed in a general purpose incubator (Model 1545, VWR International, LLC, West Chester, PA, USA) for temperature control at 48.5°C. The enzymatic hydrolysis will take a total of 7 days/168 hours. After hydrolysis, the produced slurry is centrifuged and the separated liquor are saved and refrigerated for fermentability test as described below.

### Hydrolyzate fermentablility test

The liquid phase separated from whole slurry enzymatic hydrolysis as described in the last sections were subjected to the fermentability test. Samples were analyzed using two assays developed for toxicity analysis: growth and mini-fermentation assays using *Zymomonas mobilis *8b as the biocatalyst. Toxicities were also tested for previously generated hydrolyzate liquors (after acid pretreatment with the biomass separated in the absence of saccharification enzymes). Two hydrolyzate liquor samples were also tested for toxicity for comparison purposes. The vertical reactor (VT) samples were generated from corn stover pretreated at 190°C, 3 wt % sulfuric acid for one minute in a pilot scale Sunds (Metso USA, Norcross, GA, USA) one metric ton/day vertical reactor. The conditions used to generate the horizontal reactor (HT) samples were of lower severity, 158°C, 2% sulfuric acid for five minutes in a pilot scale Metso (Metso, Norcross, GA) 200 kg/day horizontal screw reactor.

*Z. mobilis *8b was used for the evaluation of hydrolyzate toxicity. It was revived from frozen glycerol stocks for approximately 6 to 8 hrs in 10 mL of RMG (2% glucose) at 33°C prior to inoculating overnight seed cultures in RMG8X2 (8% glucose, 2% xylose) using Blank volume shake flasks filled to 80% capacity at 33°C at 120 rpm. When the glucose concentration decreased from approximately 80 g/L to approximately 20 to 40 g/L, cells were spun down at 3,840 × g for 10 minutes at room temperature and re-suspended in RMG (2% glucose, 10 g/L yeast extract, 2 g/L K_2_HPO_4_) at a 10-fold concentration and used as inocula for Bioscreen C growth assays or fermentation studies.

For growth assays, samples were diluted to 10 wt % of the starting slurry material from pretreatment with the addition of 10 g/L yeast extract, 2 g/L K_2_HPO_4_, sugars and water. Depending upon the initial concentrations of sugars present, samples were normalized to the same concentrations for glucose and xylose. In our case, concentrations of glucose and xylose are normalized to 45 g/L and 31 g/L, respectively. Pure sugar fermentations at the same concentrations were conducted as controls. Bioscreen C assays were carried out as described previously [24]. Cells were inoculated into Bioscreen C wells containing a total volume of 300 μL and incubated without shaking at 33°C at an initial cell density of OD = 0.05 (approximately 5 × 10^6 ^cells/mL). Turbidity measurements (OD_420-580 nm_) were taken every 10 minutes for up to 48 hours.

Mini-fermentation assays were conducted with whole slurries conducted at higher biomass loadings in miniature vials vented with 18 gauge needles capped with 0.2 μM filters, using a total 4 mL volume. Yeast extract, K_2_HPO_4_, water, glucose and xylose were added, while normalizing the total glucose and xylose to the same concentration for each sample. Mini-fermentations were conducted by inoculating log phase cultures of *Z. mobilis *8b at an initial cell OD of OD_600 nm _1 (approximately 1 × 10^8 ^cells/mL). Samples were taken at 0, 24, 48, and 72 hours for OD_600 nm _readings and HPLC analysis. Fermentation assays were conducted at 33°C at 120 rpm.

### Chemical analysis and yield calculations

Pretreatment and enzymatic saccharification liquors and fermentation samples were analyzed using HPLC according to standard NREL laboratory analytical procedures (LAPs) [29]. Solid residues were also analyzed according to standard NREL LAPs [29,30]. Sugar yields from high solids enzymatic hydrolysis were calculated using the equations developed by Zhu *et al. *[31].

In the liquor phase of pretreated slurry, acetate is present in two forms: 1) free acetate/acetic acid, released from the xylan; and 2) acetyl groups covalently bound to the dissolved xylan oligomers. Free acetate is measured by direct injection on a Shodex SP0810 acid column (Kawasaki, Japan), while total acetate is measured with the same column after a 4% acid hydrolysis of the filtered pretreatment liquid that contains all solubilized compounds. The 4% acid hydrolysis at 121°C for 1 h hydrolyzes the remaining acetyl groups covalently bound to the xylooligomers.

## Abbreviations

AXE: acetyl xylan esterases; INL: Iowa National Lab; LAPS: laboratory analytical procedures; PCS: pretreated corn stover; XM: xylose monomer; XO: xylose oligomer; HT: horizontaol reactor; VT: vertical reactor.

## Competing interests

The authors declare that they have no competing interests.

## Authors' contributions

XC designed and conducted the experimental work including deacetylation, pretreatment and enzymatic hydrolysis. He also analyzed the data and drafted the manuscript. JS co-conducted the experiments, reviewed results and finalized the manuscript. MAF and MZ designed and performed the mini-fermentation and the toxicity test. WW and EK reviewed the manuscript. MT led and coordinated the overall project.

All authors have read and approved the final manuscript.

## Authors' information

Dr. Xiaowen Chen received his master's and Ph.D degrees in chemical engineering from the University of Maine. He is now a post-doctoral researcher in the National Renewable Energy Lab. His research interest is in process development and biochemical engineering in cellulosic ethanol.
